# Utilisation of immediate and early postpartum intrauterine contraceptive devices among adolescents in Mbale City, Eastern Uganda

**DOI:** 10.4314/ahs.v24i2.28

**Published:** 2024-06

**Authors:** Priscilla Alupo, Julius Nteziyaremye, Rose Chalo Nabirye, Lydia VN Ssenyonga, Pamella R Adongo, Joshua Epuitai, Immaculate Mbwali

**Affiliations:** 1 Department of Nursing, Faculty of Health Sciences, Busitema University, P.O Box 1460, Mbale – Uganda; 2 Department of Obstetrics and Gynaecology, Faculty of Health Sciences, Busitema University, P.O Box 1460, Mbale – Uganda

**Keywords:** Immediate and early postpartum intrauterine contraceptive devices, rapid repeat adolescent pregnancies, postpartum adolescents, key informants, Mbale City

## Abstract

**Background:**

Uganda is predominantly a young adolescent population and has a very high (74%) rapid repeat adolescent pregnancy (RRAP) rate. The utilisation of immediate and early postpartum intrauterine contraceptive devices (PPIUCD) is the most effective strategy to immensely abate the medical and social consequences associated with adolescent pregnancies.

**Objectives:**

To determine the prevalence and factors influencing the utilisation of immediate and early PPIUCD among adolescents in Mbale City, Eastern Uganda.

**Methods:**

We used a cross sectional study design with quantitative and qualitative methods. Interviewer administered pretested semi-structured questionnaire was used to interview a sample of 422 participants. Eight key informant interviews were conducted to explore the perceived enablers and barriers to use of immediate and early PPIUCD. Qualitative data was analyzed using deductive thematic analysis.

**Results:**

The prevalence of immediate and early PPIUCD utilisation was 2.4% (10/422). Inadequate adolescent knowledge, inadequate mentorship training of health care providers, equipment and infrastructure and myths and misconceptions were perceived to limit uptake of immediate and early PPIUCD.

**Conclusion:**

The prevalence of immediate and early PPIUCD utilisation was very low. In-service training of health care workers and effective counseling of adolescents may correct the perceived myths and misconceptions thus increasing uptake of immediate and early PPIUCD.

## Background

Uganda is predominantly a young adolescent population^1^,^2^. Consequently, Uganda grapples with adolescent health related concerns^3^,^4^. Coupled with lack of sexual and reproductive health education, Uganda has high rates of early adolescent sexual debut (10.4%), adolescent pregnancies (25%), and high rapid repeat adolescent pregnancies (RRAP) (74%)^5^–^7^. Adolescent pregnancies predisposes adolescents to high risk of maternal mortality from pre-eclampsia, obstructed labor, and preterm birth^3^,^8^. Worse still, adolescent pregnancies impair the ability of girls to return and complete school which further reduces their opportunities to acquire decent employment and income of living^9^,^10^. In Uganda, adolescents contribute about 17% of the national maternal deaths^6^.

The uptake of modern contraceptives is low in Uganda with only 39% among married women and 28% of married women have unmet need for modern contraceptives^6^. Consequently, the current use of modern contraceptives indicates that Uganda may not have attained the target of increasing the uptake of modern contraceptives to 50% in 2020^4^ and the Sustainable Development goals' target to scale up the uptake of modern contraceptives by 2030 (11). Despite the low uptake of modern contraceptives, almost half (45%) of women abandon contraceptives in the first year^12^,^13^.

Immediate and early PPIUCD provides long-term contraception as it is not user dependent, does not suffer from challenges of inconsistencies and has been proven to curtail RRAP subsequently reversing maternal and child mortality and morbidity thus spurring economic development^10^,^13^–^18^. Although the utilisation of intrauterine contraceptive devices (IUCD) is generally low (4%) in Uganda, the use of IUCD is even worse in the immediate and early postpartum period^6^. Studies have noted discussion with male partners about contraceptionse as determinants influencing uptake of IUCD among adolescents^19^,^20^. Myths and misconceptions have been noted to limit the use of IUCD among adolescents^21^,^22^. Studies in Uganda have noted low uptake of modern contraceptives among adolescents^6^,^23^,^24^. Few studies have explored the determinants of the uptake of immediate and early PPIUCD (16). The study was therefore conducted to determine the prevalence and factors influencing the utilisation of immediate and early postpartum intrauterine contraceptive devices among adolescents residing in Mbale City, Eastern Uganda.

## Methods

### Study design, setting and participants

A mixed-methods study was used. Quantitative method was used to determine the prevalence and factors associated with uptake of immediate and early PPIUCD while qualitative interviews were carried out to explore in-depth information regarding the perceived barriers to uptake of immediate and early PPIUCD.

The study was conducted in four sites: Mbale Regional Referral Hospital (MRRH), Namatala Health Centre IV (HCIV), Marie Stopes-Mbale Branch and Mbale District Health Office. These health facilities are located in Mbale City in Eastern Uganda. The City has a population of 86,200 people^25^,^26^. MRRH serves over four million people within 16 districts of its catchment area (4) while Namatala HCIV serves as the top health facility under the Mbale City local administration. MRRH, Namatala HCIV and Marie Stopes offer obstetrics and Gynaecology services including family planning services.

The adolescents for quantitative interviews were within one year postpartum and were recruited from Young Child Clinics (YCC) in the health facilities. Qualitative data was collected from health care workers who were directly providing family planning services to adolescents from the postnatal wards, family planning and adolescent clinics.

### Sampling techniques and sample size

Consecutive sampling was used to recruit postpartum adolescents. Very ill postpartum adolescents and non-residents of Mbale City were excluded. Leslie and Kish formula (1965); N = Z2PQ / d2 was used to arrive at a population of 422 participants based on a prevalence assumed at 50% and a non-response rate of 10%. Purposive sampling was used to recruit eight key informants (KI) from the four study sites with guidance of the health facility in-charges.

### Data collection and study variables

Data was collected in the months of October and November 2021. Quantitative data collection was done using interviewer administered pretested semi-structured questionnaires. The dependent variables included questions on socio-demographics (age, marital status, residence, religion, highest education level and occupation), adolescent- parent / guardian communication (discussion about contraception with parent and contraception method discussed), obstetric factors (parity, planned latest pregnancy, and mode of delivery), gynaecological factors (age of sexual debut, major source of contraception information, postpartum contraception used and current use of immediate and early PPIUCD), health facility related factors (attended antenatal contacts, counseled about postpartum contraception use) and male partner's factors (age, highest education level, occupation and discussion about contraception use). The dependent variable was the utilisation of immediate and early PPIUCD among adolescents. Immediate and early PPIUCD utilisation was taken as IUCD use within first ten minutes to forty-eight hours following childbirth. KI interview guide was used to explore the perceived barriers of immediate and early PPIUCD uptake. The guide included questions on knowledge about immediate and early PPIUCD, enablers and barriers to the uptake of immediate and early PPIUCD. The interviews were conducted in English, Luganda and Lugisu the local dialects in this setting.

### Ethical considerations

Ethical approval was obtained from MRRH Research and Ethics Committee (Reference number: MRRH-2021-96). Administrative permission to conduct the study was obtained from the respective health facility in-charges. Participation in the study was voluntary. Written informed consent for emancipated minors (those aged 15-19 years) and assent (those aged 10-14 years) were obtained prior to data collection as per the principles of Uganda National Council of Science and Technology (27). Donning of face masks and hand sanitizing was practiced during interviews to prevent the spread of COVID-19. Numbers were assigned to each participant and interviews were held in private. All participants received extra information about immediate and early PPIUCD.

### Data analysis

Stata version 15.0 was used for quantitative data analysis. Categorical variables were summarized as percentages and continuous variables as means (standard deviations). Bivariate logistic regression was conducted and presented as crude odds ratios (COR), 95% confidence intervals and p values. Variables whose level of statistical significance set at p value <0.2 at bivariate analysis and plausible variables revealed to be associated with the utilisation of immediate and early PPIUCD from literature were then run for multivariate logistic regression. The significance of association from the multivariate analyses were presented as adjusted odds ratios (AOR), 95% confidence intervals and p values. Independent variables with a p value <0.05 were significantly associated with the utilisation of immediate and early PPIUCD.

Nvivo version 16 was used for qualitative data analysis where the data was categorized into themes, sub-themes and codes as per Braun and Clarke thematic analysis (28). The data was then presented in form of quotations and paraphrases of the participants' ideas.

## Results

### Quantitative results

#### Socio-demographic characteristics and adolescent-parent or guardian communication

Almost all 99.3% (419/422) of participants were aged 15-19 years. Parents or guardians discussed about the use of contraception with their adolescents in only 7.8% (33/422) of the participants. IUCD was the least 9% (3/33) contraception method that was mentioned by the parents, while some parents 15.1% (5/33) disapproved contraception use by the adolescents. (Table 1)

**Table 1:** Socio-demographic characteristics and adolescent-parent or guardian communication

Variable	Frequency (N=422)	Percentage (%)
**Socio-demographic characteristics**		
**Age (years)**		
10-14	03	0.7
15-19	419	99.3
**Marital status**		
Married	347	82.2
Not married	75	17.8
**Residence**		
Urban	352	83.4
Rural	70	16.6
**Religion**		
Roman Catholic	168	39.8
Anglican	149	35.3
Muslim	105	24.9
**Highest education level**		
Primary	298	70.6
Post-primary	124	29.4
**Occupation**		
Employed	99	23.5
Unemployed	323	76.5
**Adolescent- parent or guardian communication**		
**Discussed about contraception with parent**		
Yes	33	7.8
No	389	92.2
**Contraception method discussed (N=33)**		
IUCD use	03	9.1
Other contraceptives use	25	75.8
Discouraged against use	05	15.1

**N**: Number of participants; **Post- primary**: Secondary and Tertiary; **Unemployed:** Currently schooling, housewives; **Other contraceptives:** Injectable contraceptives, oral pills, implants.

#### Obstetric ad Gynaecological characteristics of participants

Majority 80.6% (340/422) never planned their most recent pregnancy. About half 49.8% (210/422) revealed health workers being their major source of contraception information and minority 2.4% (10/422) were currently using immediate and early PPIUCD. (Table 2)

**Table 2:** Obstetric and Gynaecological characteristics of participants

Variable	Frequency (N=422)	Percentage (%)	Mean (SD)	Min	Max
**Obstetric characteristics**					
**Parity**					
Primiparity	401	95.0			
Multiparity	21	5.0			
**Planned latest pregnancy**					
Yes	82	19.4			
No	340	80.6			
**Mode of delivery**					
Vaginal delivery	370	87.7			
Caesarean delivery	52	12.3			
**Gynaecological characteristics**					
**Age of sexual debut (years)**			16.3 (±1.4)	10	19
<16	105	24.9			
≥16	317	75.1			
**Major source of contraception**					
**Information**					
Health workers	210	49.8			
Other sources	194	45.9			
Never heard about	18	4.3			
contraception					
**Postpartum contraception used**					
IUCD	13	3.1			
Other contraceptives	88	20.8			
None	321	76.1			
**Currently using immediate and early PPIUCD**					
Yes	10	2.4			
No	412	97.6			

**Other sources:** Electronic media (radios, television, internet), school, friends and relatives; **Min**: Minimum; **Max:** Maximum.

#### Health facility related and male partners' characteristics

Less than half 46.0% (187/407) admitted to being counseled about postpartum contraception use of which only 44.4% (83/187) were counseled about immediate and early PPIUCD. Only 34.6% (146/422) had ever discussed with their spouses about postpartum contraception use. (Table 3)

**Table 3:** Health facility related and male partners' characteristics

Variable	Frequency (N=422)	Percentage (%)	Mean (SD)	Min	Max
**Health facility related characteristics**					
**Attended antenatal Contacts**					
Yes	407	96.5			
No	15	3.5			
**Counseled about postpartum Contraception**					
**use (N=407)**					
Yes	187	46.0			
No	220	54.0			
**Counseled about immediate and early**					
**PPIUCD (N=187)**					
Yes	83	44.4			
No	104	55.6			
**Male partners' characteristics**					
**Age (years)**			24.3 (±4.3)	13	36
≤19	66	15.6			
≥20	356	84.4			
**Highest education level**					
Primary	187	44.3			
Post-primary	235	55.7			
**Occupation**					
Employed	382	90.5			
Unemployed	40	9.4			
**Discussed about contraception use**					
Yes	146	34.6			
No	276	65.4			

Prevalence of utilisation of immediate and early postpartum intrauterine contraceptive devices among adolescents Only 2.4% (10/422) of the adolescents were currently using immediate and early PPIUCD. Because of the small proportion who were using immediate and early PPIUCD, logistic regression to determine the factors associated with utilization of immediate and early PPIUCD was not conducted.

### Qualitative results

#### Socio-demographic characteristics of key informants

There were five female and three male key informants (KI) aged 28-48 years. The KI interviewed included one doctor, five nurses and two midwives. Each KI had a working experience of at least three years.

#### Perceived barriers to uptake of PPIUCD among adolescents

##### Inadequate counselling about immediate and early PPIUCD uptake

The KI perceived that the barrier to immediate and early PPIUCD use among adolescents was related to inadequate knowledge about the method because of missed antenatal care attendance.

*“Most health education about IUCD use is given during antenatal visits. However, the challenge is that adolescents do not attend the recommended number of antenatal contacts and so miss out on being counseled about IUCD use”* (KI 1 a 28year old female nurse with 5 years of working experience).

*“Adolescents are not taught about family planning as part of their curriculum in schools. Most of them actually learn from their peers and relatives, so they end up getting fragmented information about family planning methods”* (KI 5 a 37 year old female midwife with 8 years of working experience).

##### Lack of skills, equipment and infrastructure

The KI revealed that their experience on insertion of IUCD was hindered by lack of in-service training on insertion of IUCD, inadequate funding allocated for IUCD requirements and inadequate infrastructure needed for insertion of IUCD.

*“We haven't had any refresher course training about insertion of IUCD ever since I started working in this clinic. My skills are based on what I learned from school only”* (KI 8 a 34year old male nurse with 4 years of working experience).

*“Majority of the adolescents we receive here prefer to use the injectable contraceptives and implants only. This has made it challenging for us to get the hands- on experience on insertion of IUCD. Also; after these mothers give birth, they focus more on the care of their newborns and neglect the use of family planning until after they get their first menses”* (KI 4 a 27year old male nurse with 3years of working experience).

*“We have experienced expiry of the IUCD materials on multiple occasions because we have very few adolescents who opt to use IUCD and so we end up discarding them. This has forced us to actually reduce on the quantity of IUCD materials stocked in”* (KI 5 a 37year old female midwife with 8years of working experience).

*“Our facility does not have a room designated for insertion of IUCD and so we have to improvise by using the postpartum ward rooms for insertion of IUCD”* (KI 7 a 31year old female midwife with 7 years of working experience).

##### Myths and misconceptions about PPIUCD

KI revealed that the beliefs, experiences and misconceptions of adolescents have limited the uptake of immediate and early PPIUCD.

*“Adolescents believe that IUCD damages the uterus by causing wounds and scars leading to infertility. They also say that that IUCD causes endometrial cancer and these have made them fear to use this method”* (KI 2 a 32year old male doctor with 6 years of working experience).

*“Some adolescents report that their religious sect forbids them from using any family planning method because it is a form of preventing child bearing”* (KI 3 a 48year old female nurse with 10+ years of working experience).

*“Most adolescents think that IUCD is a contraceptive method for the older women of reproductive age who have already birthed many children and it is not appropriate for them who are still young and plan on giving birth to children soon”* (KI 6 a 46year old female nurse with 10+ years of working experience).

##### Lack of male partner support

KI perceived that male partners' participation in decision making process influenced the utilisation of immediate and early PPIUCD.

*“Some adolescents report that their spouses refuse them from using IUCD because it reduces pleasure during sexual intercourse because of the IUCD strings hanging within the internal genitalia”* (KI 2 a 32year old male doctor with 6 years of working experience).

*“Male partners don't consent to the use of IUCD because it is a contraceptive method that lasts about ten to twelve years which is a very long time of preventing pregnancy yet they need to produce children within an interval of about two to five years. They prefer their women to use injectable contraceptives and implants which are shorter lasting”* (KI 3 a 48 year old female nurse with 10+ years of working experience).

## Discussion

The study was conducted to determine the utilisation of immediate and early PPIUCD among adolescents in Mbale City in Eastern Uganda. The uptake of immediate and early PPIUCD among adolescents were 2.4%. The perceived barriers for the low uptake of immediate and early PPIUCD were inadequate counseling, skills, equipment and infrastructure needed for PPIUCD insertion, myths and misconceptions about PPIUCD and lack of male partner support. The findings of this study have critical implications for uptake of PPIUCD among the vulnerable population.

This study revealed that 2.4% of the adolescents were currently using PPIUCD in the immediate and early post-partum period. This is consistent with the low uptake of 3.4% in Kenya (29) and 4.0% in Ethiopia^30^. However, our study was not comparable to findings in other settings which found significantly higher prevalences of 26.6% (19), 28.1% (16) and 35.6%^31^. Unlike other contraceptive methods that are plagued with problems of inconsistent use, high failure rates, and likelihood of early discontinuation, PPIUCD offers long term contraceptive effect reducing rapid repeat adolescent pregnancy^10^,^13^–^18^. The low uptake of immediate and early PPIUCD therefore underscores scholarly and policy implications that would promote scale up and adoption of immediate and early PPIUCD among the high-risk adolescent population.

Our exploration study revealed that a majority of the adolescents are not informed about the benefits of immediate and early PPIUCD because of irregular attendance of antenatal contacts and so miss out on being counseled about the use of early and immediate PPIUCD. Worse still, parental or guardian discussion about PPIUCD use with their adolescents has been shunned which has deprived adolescents of this vital information. Several studies have noted regular attendance of antenatal contacts^16^,^31^ and parental guidance on sexual and reproductive health concerns^3^,^20^,^32^ as positive influencers to the high uptake of immediate and early PPIUCD. Therefore, emphasis on regular antenatal attendance and parental or guardian involvement in discussion with adolescents about their sexual and reproductive health concerns may increase the uptake of immediate and early PPIUCD in our study area.

Our qualitative study findings emphasized lack of in-service training of health care providers and inadequate PPIUCD equipment and infrastructure in terms of rooms for insertion of PPIUCD as inhibitors to the provision of immediate and early PPIUCD services to adolescents. Other studies attributed the high uptake of immediate and early PPIUCD to training of the health care providers prior to insertion of PPIUCD and availability of PPIUCD equipment^16^,^32^. Therefore, in-service mentorship training of health care providers should be advocated to increase uptake of immediate and early PPIUCD among adolescent girls in our study setting.

Our exploration study revealed that PPIUCD was perceived to cause endometrial cancer and infertility; misconceptions which provided a strong emotive barrier to uptake of immediate and early PPIUCD. Consistent findings have been revealed in other studies^21^,^22^. Additionally, the preference for short acting contraceptives escalated the low uptake of immediate and early PPIUCD. Therefore, mass health education and counseling should be adopted to correct the myths and misconceptions about immediate and early PPIUCD in order to increase uptake and as well reduce the rampant rapid repeat adolescent pregnancies brought about by inconsistencies of short acting contraceptives and unmet need for immediate and early PPIUCD.

Previous studies have shown that male involvement positively influences uptake of modern family planning methods^1^,^33^–^35^. This was similar to our qualitative findings where lack of male partner approval was perceived as a barrier to adoption of immediate and early PPIUCD. However, reliance on male partners on decision making about use of immediate and early PPIUCD in our study setting with deeply entrenched patriarchal systems and widespread gender inequalities underscores the role of male partners in decision making regarding the uptake of immediate and early PPIUCD. Therefore, having a male partner buy-in will be key in unlocking the potential for adolescent girls to use immediate and early PPIUCD.

## Conclusion

The prevalence of utilisation of immediate and early PPIUCD among adolescents was 2.4%. The barriers to uptake of immediate and early PPIUCD were perceived as inadequate counseling about immediate and early PPIUCD, inadequate skills of health care providers, deficient equipment and infrastructure, myths and misconception and lack of male partner approval. Regular antenatal attendance, parental or guardian discussion with adolescents, in-service training of health care providers, adequate counseling and provision of PPIUCD equipment and infrastructure will drastically improve uptake of immediate and early PPIUCD.

## Study strength and limitations

Our study used a facility based mixed- method approach. The qualitative findings explored the perceived barriers to the utilisation of immediate and early PPIUCD. Our study was not statistically powered enough to determine the factors associated with immediate and early PPIUCD uptake among adolescents given the small proportion of immediate and early PPIUCD uptake.
